# The Treatment of Adult Bipolar Disorder with Aripiprazole: A Systematic Review

**DOI:** 10.7759/cureus.562

**Published:** 2016-04-07

**Authors:** Ather Muneer

**Affiliations:** 1 Psychiatry, Islamic International Medical College, Rawalpindi,Pakistan

**Keywords:** bipolar disorder, bipolar mania, bipolar depression, mood stabilizers, third generation antipsychotics

## Abstract

Bipolar disorder is characterized by exacerbations of opposite mood polarity, ranging from manic to major depressive episodes. In the current nosological system of the Diagnostic and Statistical Manual – 5^th^ edition (DSM-5), it is conceptualized as a spectrum disorder consisting of bipolar disorder type I, bipolar disorder type II, cyclothymic disorder, and bipolar disorder not otherwise specified. Treatment of all phases of this disorder is primarily with mood stabilizers, but many patients either show resistance to the conventional mood stabilizing medications or are intolerant to their side-effects. In this setting, second-generation antipsychotics have gained prominence as many bipolar subjects who are otherwise treatment refractory show response to these agents. Aripiprazole is a novel antipsychotic initially approved for the treatment of schizophrenia but soon found to be effective in bipolar disorder. This drug is well studied, as randomized controlled trials have been conducted in various phases of bipolar disorders. Aripiprazole exhibits the pharmacodynamic properties of partial agonism, functional selectivity, and serotonin-dopamine activity modulation – the new exemplars in the treatment of major psychiatric disorders. It is the first among a new series of psychotropic medications, which now also include brexpiprazole and cariprazine. The current review summarizes the data from controlled trials regarding the efficacy and safety of aripiprazole in adult bipolar patients. On the basis of this evidence, aripiprazole is found to be efficacious in the treatment and prophylaxis of manic and mixed episodes but has no effectiveness in acute and recurrent bipolar depression.

## Introduction and background

Bipolar disorder (BD) is a common neuropsychiatric condition with a prevalence rate of approximately 4% in the general population, considering the entire bipolar spectrum [1]. It is characterized by severe disturbances in the mood of opposite polarity, which range from mania to depression. It is a chronic condition with the usual onset in adolescence or early adulthood and affects males and females equally [2]. It is highly comorbid with other diseases, such as anxiety spectrum disorders, substance abuse disorders, eating disorders, stressor-related disorders, and certain personality disorders, chiefly borderline personality disorder [3]. It can be complicated by severe depressive exacerbations, mixed episodes, rapid cycling, and subthreshold symptoms and is associated with one of the highest suicide rates among all psychiatric disorders [4]. Even after adequate treatment with currently available agents, affective episodes often fail to remit completely with persistence of symptoms and accompanying myriad complications in the biopsychosocial domain. 

**Figure 1:**
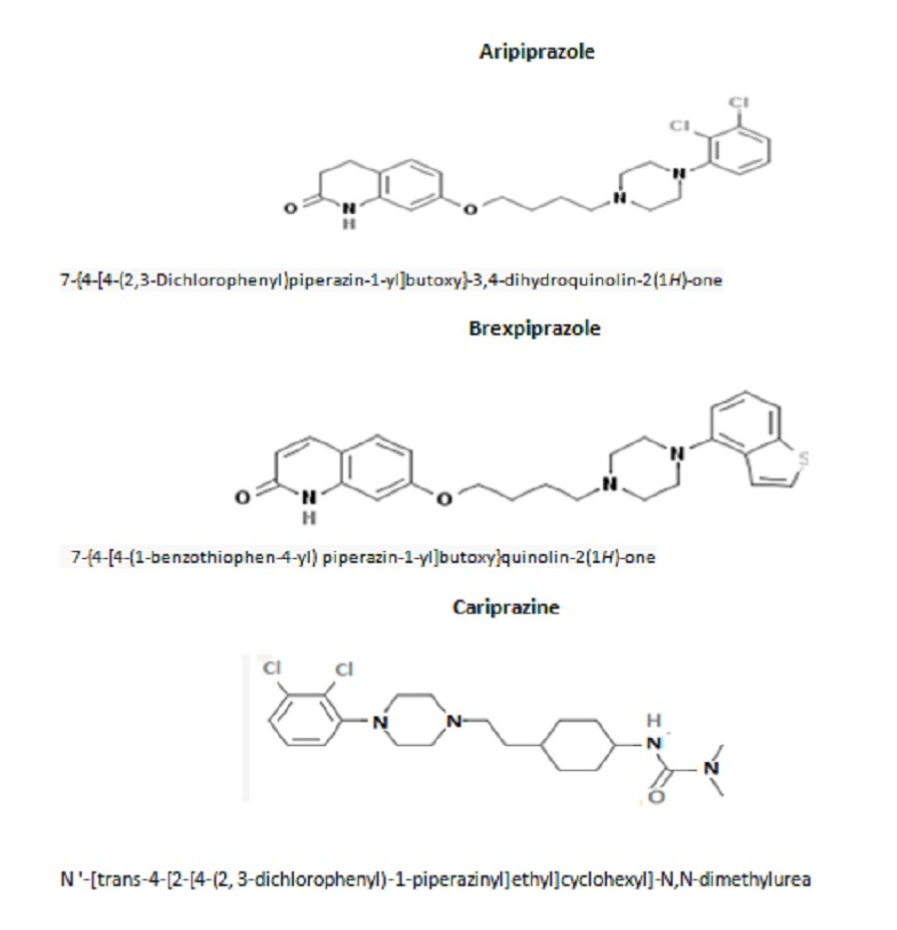
The chemical structures and IUPAC names of aripiprazole, brexpiprazole and cariprazine IUPAC – International Union of Pure and Applied Chemistry nomenclature

In this event, there is an unmet need for medications that are truly effective in different phases of the disorder. Unlike conventional mood stabilizers and older antipsychotics, second-generation antipsychotics (SGA) are multifunctional as these have shown efficacy in a broad range of psychiatric disturbances, including manic, depressive, and maintenance phases of bipolar disorder [5]. SGAs are exemplified by medications, such as risperidone, olanzapine, quetiapine, ziprasidone, and lurasidone, which are widely employed in the treatment of BD [6]. In addition to these, a new group of agents, the prototype of which is aripiprazole, is also available. The armamentarium of medications in this regard has increased with the United States Food and Drug Administration (FDA) approval of brexpiprazole and cariprazine. These are related compounds, and Figure 1 gives their chemical structures for the sake of comparison.

These novel agents have been variously termed “third generation antipsychotics” or “serotonin-dopamine activity modulators” and are gaining prominence in the treatment of major psychiatric ailments, such as BD, major depressive disorder (MDD), and schizophrenia. There are subtle but important differences in the mechanism of action of SGAs and novel antipsychotics. Whereas the former act as full antagonists or inverse agonists at the dopamine D2 receptor (D2R), the latter behave as partial agonists at this site [7]. They may have disparate activity in different areas of the brain, depending on the prevalent dopaminergic tone, and act as full antagonists in regions with high dopamine neurotransmission but as partial agonists where this trend is low [8]. Presumably, this dual action has a putative mitigating effect on positive psychotic symptoms by virtue of the dopamine-blocking action in the mesolimbic area, whereas there is alleviation of negative and cognitive symptoms because of the facilitation of dopamine transmission in the prefrontal cortex [9]. Further, novel antipsychotics purportedly act as biased ligands at the D2R, which is a G-protein-coupled receptor (GPCR). This implies that they differentially stimulate intraneuronal signal transduction pathways, in contradistinction to other antipsychotics, which do not discriminate with downstream signaling cascades. This phenomenon has been termed “functional selectivity” and may have key connotations in the treatment of main psychiatric conditions by novel antipsychotics [10].

In the case of BD, repeated affective episodes have been shown to cause neuroinflammation, oxidative, and nitrosative stress leading to increased apoptosis of neurons in the hippocampus and other key mood-regulating areas of the brain. Administration of psychotropic drugs, like the novel antipsychotics, is neuroprotective with dendritic growth, increased synaptogenesis, and enhanced viability of neurons [11]. With the continued use of these medications, the deteriorating course of BD, a fundamentally neuroprogressive condition, is halted and is clinically manifested as longer periods of euthymia and improved psychosocial functioning of the patients.

Aripiprazole, the forerunner of the novel antipsychotics, has been well studied in different phases of BD. This review presents a comprehensive appraisal of the extant literature, focusing on randomized controlled trials (RCT). This endeavor illuminates the role of aripiprazole in the treatment of adults with BD, a difficult to treat condition, which manifests in innumerable ways and is often refractory to the currently available psychotropic agents.  

## Review

### Search strategy

**Figure 2:**
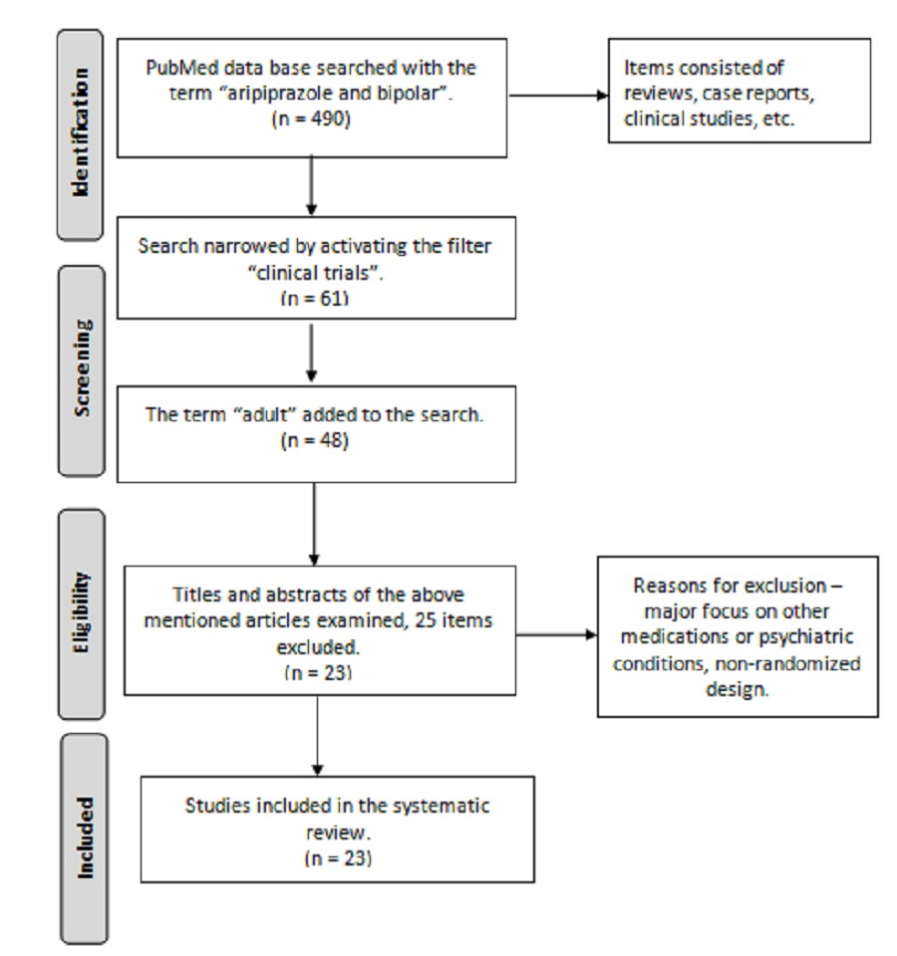
Flow diagram of the systematic review Selection of the studies according to the PRISMA statement. PRISMA - Preferred Reporting Items for Systematic Reviews and Meta-analyses.

A search of the PubMed database was conducted in October 2015 with the inclusive term “aripiprazole and bipolar.” A total of 490 items were found which included reviews, case reports, pharmacokinetic investigations, clinical and preclinical studies. Narrowing the search to “clinical trials” retrieved 61 items and further refinement with “adults” resulted in 48 studies. The titles and abstracts of these papers were examined for relevance to the current topic, leading to exclusion of a further 25 studies. The rationale was to leave out records that had their main focus on other medications or psychiatric conditions. The resulting 23 studies were read in full and their reference lists consulted. Data from these papers was used to mark out the place of aripiprazole in the treatment of adults with bipolar disorder. Figure 2 illustrates the study selection process according to the PRISMA (Preferred Reporting Items for Systematic Reviews and Meta-analyses) statement. Table 1 lists the studies included in the systematic review.

**Table 1:** Studies Evaluating Efficacy and Safety of Aripiprazole in Different Phases of Bipolar Disorder AE – adverse events; ARI – aripiprazole; BD – bipolar disorder; DB – double blind; CGI-BP – Clinical Global Impressions scale- Bipolar Version; EPS –extrapyramidal symptoms; HRSD – Hamilton Rating Scale for Depression; HPD – haloperidol;  LI –lithium; LOCF – last observation carried forward; LTG – lamotrigine; MADRS – Montgomery-Asberg depression rating scale; MS – mood stabilizer; NNT – number needed to treat; NNH – number needed to harm; PLB – placebo; RCT – randomized controlled trial; VAL – divalproex sodium; YMRS – Young Mania Rating Scale.

Study	Type	Methodology	Result
STUDIES IN ACUTE MANIA			
Kanba, et al., 2014 [27]	RCT	ARI (dosed from 12-24 mg/d, majority receiving 24 mg/d) compared to PLB in this 3-week, acute phase study. Primary efficacy measure - change in YMRS score from baseline to endpoint at day 21.	ARI significantly more effective than PLB with early separation on YMRS observed from Day 4. In the ARI group, no significant metabolic derangement or prolactin elevation detected compared to PLB.
Jeong, et al., 2012 [32]	Single blind design	Acutely manic patients randomized to ARI + VAL or HPD + VAL and followed for 8 consecutive weeks. YMRS and CGI-S used to assess efficacy. AE monitored with Drug-induced Extrapyramidal Symptoms Scale and Liverpool University Neuroleptic Side Effect Rating Scale.	Both groups had similarly high response and remission rates. HPD associated with more EPS, while adjunctive ARI group had greater weight gain and sedative side effects.
El Mallakh, et al., 2010 [28]	RCT	This 3-week trial conducted in BD Type I hospitalized patients with acute manic or mixed episodes. Subjects randomized to ARI, 15 mg/d, ARI, 30 mg/d, or PLB. Primary outcome parameter, change in YMRS score from baseline to day 21.	ARI, both doses not significantly superior to PLB on the main efficacy measure. Compared to PLB, ARI group had notably more headache, agitation, and akathisia.
Findling, et al., 2009 [33]	RCT	Patients aged 10 to 17 years with acute manic or mixed episodes (with or without psychosis) randomized to ARI, 10 mg, ARI, 30 mg, or PLB. Subjects followed for 4 weeks and primary efficacy measure was change in YMRS score from baseline.	ARI (both doses) significantly superior to PLB on global improvement and overall bipolar illness outcome measures. Most common AE were EPS and somnolence. Average weight gain was not significantly different among the three groups.
Keck, et al., 2009 [30]	RCT	A PLB and LI-controlled study, primary endpoint at Week 3. ARI and LI administered patients remained on blinded treatment for 9 additional weeks. Subjects included Bipolar I patients with acute manic or mixed episodes (YMRS ≥ 20) with or without psychotic features. Primary efficacy measure – change in baseline to Week 3 on YMRS for the entire study population. Secondary outcome – change in YMRS to Week 12 between ARI and LI groups.	Both ARI and LI significantly better than PLB on primary outcome. ARI and LI equally effective in controlling manic symptoms. ARI associated with akathisia and headache and LI with GI side effects and tremor during the trial.
Young, et al., 2009 [29]	RCT	ARI compared to HPD and PLB for the first stage of the trial (3 weeks). ARI evaluated against HPD for the next phase - to Week 12. YMRS employed as the psychometric instrument to assess efficacy.	ARI and HPD notably better than PLB. Both drugs maintained efficacy to Week 12. ARI superior to HPD in tolerability with fewer EPSE.
Vieta, et al., 2008 [21]	RCT	Bipolar I disorder patients, current episode manic or mixed were treated with LI or VAL monotherapy. Those with non-response (YMRS ≥ 16) with therapeutic blood levels, randomized to adjunctive ARI or PLB and followed up for 6 weeks. Primary efficacy measure change in YMRS score to Week 6.	Adjunctive ARI+MS, significantly better than MS monotherapy on main outcome with separation occurring on YMRS from Week 1 onwards. Akathisia significantly more common with ARI than PLB.
Suppes, et al., 2008 [34]	Post-hoc analysis	Data pooled from two 3-week, flexible-dose, RCTs in BD Type I patients with index manic or mixed episodes. The patient population stratified by severity of episode (YMRS score), baseline depressive symptoms (MADRS score), and presence or absence of psychotic features/rapid cycling.	ARI significantly better than PLB in patients groups with more or less severe illness, manic or mixed episodes, with or without psychotic features and rapid cycling. ARI effective in a wide array of disease characteristics associated with treatment resistance.
Sachs, et al., 2006 [20]	RCT	BD Type I patients with manic or mixed episodes randomized to ARI, 30 mg/d, or PLB and studied for 3 weeks. Primary efficacy measure was change in baseline YMRS to endpoint. Secondary assessment was with CGI-BP.	ARI significantly better than PLB in producing response, defined as ≥ 50% reduction in YMRS. ARI, 30 mg/d, well tolerated by 85% of the patients. ARI and PLB with similar profiles on metabolic parameters, QTc interval, and serum prolactin.
Vieta, et al., 2005 [31]	DB design.	Bipolar I patients with acute manic or mixed episodes randomized to ARI or HPD and followed up for 12 weeks. Primary outcome measure was number of patients in response (≥ 50% reduction in YMRS) and receiving treatment at endpoint.	Significantly more subjects in the ARI group as compared to HPD group showed response and were on treatment at Week 12. Greater discontinuation rate in the HPD group, with higher rates of EPS.
Keck, et al., 2003 [19]	RCT	Patients with acute manic or mixed episodes randomized to ARI, 30 mg/d, or PLB. Primary outcome - mean change in total YMRS score from baseline to endpoint. Secondary outcome with CGI-BP.	ARI significantly better than PLB on primary and secondary efficacy measures with changes observed as early as Day 4. ARI produced higher response and completion rates. ARI and PLB were statistically similar with regards to metabolic changes, QTc interval effects, and prolactin levels.
CONTINUATION PHASE STUDIES IN PATIENTS WITH INDEX MANIC OR MIXED EPISODE			
Yatham, et al., 2013 [37]	Post-hoc analysis	In this study, data from a 52-week RCT was examined with regards to relapse prevention in bipolar patients with index manic or mixed episodes. Cases were initially stabilized with single-blind ARI + LI or VAL; those maintaining stability for 12 weeks were randomized to ARI + LI or VAL or PLB + LI or VAL for up to 52 weeks in DB fashion.	Pooled data showed that adjunctive ARI significantly superior to PLB in relapse prevention in patients with manic but not mixed episodes. ARI + MS produced significant reductions in YMRS scores in both populations. Adjunctive ARI may be a more appropriate maintenance treatment for bipolar subjects with recurrent manic episodes.
Carlson, et al., 2012 [40]	RCT	Adjunctive ARI with LTG compared to LTG monotherapy in relapse prevention in BD I patients. Subjects with recent episode manic or mixed were stabilized with ARI + LTG (YMRS/MADRS ≤ 12) in non-DB fashion for 9 to 24 weeks. Cases controlled for 8 consecutive weeks were randomized to ARI + LTG or PLB + LTG, DB for 52 weeks total.	ARI + LTG not statistically superior to LTG alone, in delaying time to manic or mixed relapse. NNT/NNH ratio was 9/22. Akathisia, anxiety, and insomnia more frequent in the ARI + LTG versus PLB + LTG group.
El-Mallakh, et al., 2012 [41]	DB design.	Forty week continuation of a 12 week study comparing ARI and LI monotherapy in Bipolar I patients, current episode manic or mixed. Cases who maintained stability (YMRS ≤ 12) in the 12-week DB study, carried on in the same manner for a total of 52 weeks. Outcome parameters were mean change in total YMRS and MADRS scores from baseline to Week 52, with results reported for observed cases only.	A high drop-out rate in both groups. ARI alone and LI alone, equally effective in relapse prevention in Bipolar I patients with index manic or mixed episode. More frequent AE with ARI included akathisia, headache, somnolence, and anxiety. On metabolic parameters, no significant difference between the two treatments.
Woo, et al., 2011 [39]	RCT	BD Type I patients, current episode manic or mixed stabilized with ARI + VAL for 6 weeks in an open-label manner. Those meeting stabilization criteria followed up for 24 weeks in DB way receiving ARI + VAL or PLB + VAL.	Relapse rate less with adjunctive ARI than with VAL monotherapy, but this observation did not reach statistical significance. No serious adverse events noted with ARI + VAL combination.
Marcus, et al., 2011 [22]	RCT	BD Type I patients, recent episode manic or mixed treated with open-label LI or VAL for at least 2 weeks. Non-responders (YMRS ≥ 16) administered adjunctive ARI in non-DB fashion. Those achieving stabilization with this regimen (YMRS and MADRS ≤ 12) for 12 successive weeks randomized to ARI + LI or VAL or PLB + LI or VAL, DB and followed up for 52 weeks.	Adjunctive ARI + MS significantly better than MS monotherapy in delaying time to any relapse. AE more common in the ARI group were tremor, headache, insomnia, and weight gain.
Vieta, et al., 2010 [38]	Open-label continuation of acute phase adjunctive ARI versus PLB study.	Completers of a 6-week DB ARI + LI or VAL versus PLB + LI or VAL trial could continue with open-label ARI + LI or ARI + VAL for further 46 weeks. The purpose of the study was to compare the efficacy, safety, and tolerability of ARI + LI versus ARI + VAL in the continuation phase.	High attrition rates in the two groups. Using LOCF, both groups maintained significant improvements in total YMRS and MADRS scores during the open-label continuation period. Most frequent AEs in the two groups included tremor, akathisia, headache, insomnia, and weight gain.
Keck, et al., 2006 [36]	RCT	BD I patients current episode manic or mixed and hospitalized for treatment were stabilized (YMRS ≤ 10/MADRS ≤ 13 for 6 successive weeks) with open-label ARI, 15 or 30 mg/d. They were subsequently randomized to ARI or PLB, DB for the total study duration of 26 weeks. Primary endpoint defined as discontinuation due to precipitation of any episode – manic, mixed, or depressive.	ARI, 15 or 30 mg/d, significantly better than PLB in prolonging time to manic but not depressive relapse. Adverse effects (reported in ≥ 5% of patients and twice the rate of PLB) were akathisia, tremor, extremity pain, and vaginitis. Weight gain occurred in ARI monotherapy group (13%) but not in the PLB group.
MAINTENANCE PHASE STUDIES			
Keck, et al., 2007 [23]	RCT	Firstly, patients with index manic or mixed episodes were stabilized (YMRS ≤ 10, MADRS ≤ 13 for 6 consecutive weeks) with open-label ARI (15 or 30 mg/d). Stabilized patients were randomized to ARI or PLB, DB and followed for 26 weeks. Cases who maintained remission at the end of this period were studied for further 74 weeks (total duration 100 weeks) in a similar manner.	Cases in remission inducted in the extension phase and treated with ARI showed a statistically significant longer time to manic relapse but not to depressive relapse. AE in ARI group observed in ≥ 5% of the subjects and twice the rate of PLB were tremor, akathisia, dry mouth, hypertension, weight gain, vaginitis, abnormal thinking, pharyngitis, and flu syndrome. The weight increase in the ARI treated patients (LOCF) over the 100 weeks was of small magnitude + 0.4 (range: +/- 0.8) kg.
Muzina, et al., 2008 [42]	Post-hoc analysis	Rapid cycling BD is difficult to treat as continued remission is hard to achieve. In the 100 week maintenance study, rapid cycling patients were identified (total = 28, ARI = 14, PLB = 14). Twelve (ARI = 7, PLB = 5) completed the 26-week phase. Only 3 finished the 100 week period (all ARI group).	In rapid cycling Bipolar I patients with index manic or mixed episode, ARI significantly superior to PLB in delaying time to relapse in both the initial and extension phases of the study. This demands further research with prospectively designed and adequately powered trials.
STUDIES IN BIPOLAR DEPRESSION			
Quante, et al., 2010 [46]	RCT	Patients with acute bipolar depression treated initially with MS (LI or VAL). Open label citalopram added and subjects randomized to ARI or PLB in DB fashion and followed up for 6 weeks. Primary efficacy measure was HRSD.	Adjunctive ARI no better than PLB on primary efficacy measure and there was no significant difference between both groups at any point. Lack of additional benefit with ARI attributable to already good effectiveness of the control group because of treatment with citalopram.
Thase, et al., 2008 [45]	RCT	Two similarly designed, multicenter, 8-week, monotherapy trials of ARI versus PLB in Bipolar I outpatients experiencing MDE without psychotic features were conducted. ARI initiated at 10 mg/d, then flexibly dosed from 5 to 30 mg/d based on clinical effect and tolerability. Primary efficacy measure was change in total MADRS score from baseline to Week 8. Secondary outcome was with CGI-BP.	At endpoint, ARI failed to separate from PLB on both primary and secondary efficacy measures. ARI group suffered from greater rates of akathisia, insomnia, nausea, fatigue, restlessness, and dry mouth. Discontinuation rates higher with ARI than PLB.
Thase, et al., 2011 [47]	Post- hoc analysis	Patients in the above-mentioned two studies were stratified according to the severity of core depressive symptoms at baseline using the Bech-Rafaelsen subscale. Bech-6 total score > 15, more severely depressed; Bech-6 total score < 15, less severely depressed. Efficacy of the active agent was evaluated employing changes in MADRS total and MADRS-6 subscale scores from baseline to Week 8.	Those classified as more severely depressed at the initial presentation had greater reduction in depression scores with ARI than PLB and this change reached statistical significance. The post-hoc analysis showed that sub-sets of patients with more severe core depressive symptoms at baseline benefited from ARI monotherapy.

### Pharmacology of aripiprazole

Pharmacokinetic Data

The drug is available in oral form and in an intramuscular formulation for acute administration for the rapid control of agitated psychotic behavior. More recently, a long-acting injectable composition has been approved by the FDA for the maintenance treatment of schizophrenia. The orally taken drug is well absorbed with a bioavailability of 87% and can be used without regards to ingestion of food. It shows linear pharmacokinetics at therapeutic doses with peak plasma concentrations reached between three to five hours after intake. Aripiprazole has a long half-life of approximately 75 hours so that patients can still maintain therapeutic blood levels even if they forget to take it in the short term [12]. Steady state plasma levels are achieved after about 14 days of continuous administration. It is extensively metabolized in the liver by cytochrome P450 (CYP) 2D6 and 3A4 isoenzymes. The former is responsible for dehydrogenation and hydroxylation and the latter for dealkylation of the drug. Dehydroaripiprazole is the active metabolite of the drug and has an even longer half-life of 96 hours. Aripiprazole does not induce or inhibit CYP450 enzymes and, therefore, causes no interference with the elimination of other medications. However, subjects who are deficient in CYP2D6 (8% of Caucasians) have about a 60% higher blood concentration of aripiprazole, and in such individuals, the half-life of the compound approximately doubles [13]. The selective serotonin reuptake inhibitor, paroxetine, is a strong inhibitor of CYP2D6 and greatly reduces the hepatic clearance of aripiprazole. Likewise, inducers (e.g., carbamazepine) or inhibitors (e.g., fluvoxamine) of CYP3A4, respectively, decrease or increase serum aripiprazole levels.

Pharmacodynamic Data – Dopamine D2 Receptor Binding

Aripiprazole is a partial agonist at dopamine D2R; it has a high affinity for this site and, at therapeutic doses, occupies approximately 95% of D2R in the striatum. However, it shows low intrinsic activity at this receptor and causes much lower activation of the D2R compared to the endogenous ligand, dopamine [14]. The key notion put forward is that it acts as a full antagonist at D2R when the synaptic concentration of dopamine is high and as a partial agonist when this value is low. Taking together its high affinity and partial agonism at D2R, it supposedly antagonizes the burst firing of dopaminergic neurons, explaining its therapeutic effects; at the same time, it allows tonic activity of this neurotransmitter, resulting in a low incidence of extrapyramidal side effects (EPSE). The consequence of this mechanism may be an overall reduction in dopamine activity in the mesolimbic region of the brain while there is a maintenance of stimulant effect in the mesocortical and nigrostriatal areas [15].

Functional Selectivity/Biased Ligand Pharmacology

**Figure 3:**
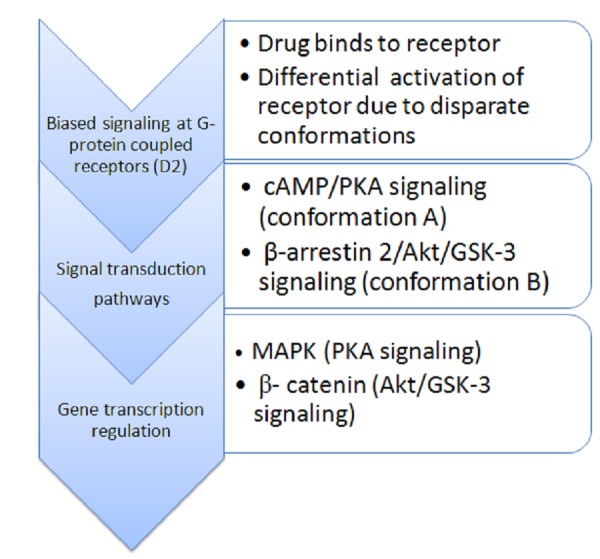
Purported mechanism of biased signaling by novel antipsychotics Biased ligands are presumed to act in a functionally discriminating manner at G-protein-coupled receptors, e.g. D2-type. One method of functional selectivity may be the favored binding to diverse conformations of the receptor, activating different downstream pathways according to the local milieu and the neuronal subtypes in which these are expressed. Postsynaptic scaffolding proteins, adaptors, and effectors may be discrepantly affected by each receptor conformation-related cascade differentially stimulated by the ligand. Akt – protein kinase B; cAMP – cyclic adenosine monophosphate; GSK-3 – glycogen synthase kinase-3; MAPK – mitogen-activated protein kinase; PKA- protein kinase A.

D2R is a G-protein-coupled receptor, and ligand binding results in downstream signaling via inhibition of adenyl cyclase. However, experiments in rodents have shown that D2R-mediated effects can occur independently of this mechanism through the scaffolding protein, β-arrestin 2. The latter associates with protein kinase B (Akt) and protein phosphatase 2A, causing activation of glycogen synthase kinase-3 (GSK-3); β-arrestin 2/Akt/GSK-3 signaling is implicated in major psychiatric disorders, including BD and schizophrenia [16]. Drugs like aripiprazole can have a differential effect on GPCR and, acting as a biased ligand, can discerningly activate one or the other pathway depending on receptor conformation. The supposed functional selectivity at GPCR by agents like aripiprazole, brexpiprazole, and cariprazine have given novel perspective to the treatment of hitherto intractable mental conditions and will undoubtedly be a principal focus for the development of better and more effective psychotropic medications [17]. Figure 3 schematically illustrates key concepts in this pharmacodynamic model.

Other Receptor Interactions of Aripiprazole

In several in vitro experiments, aripiprazole has performed as a partial agonist at 5HT1A receptors, whereas other data suggest that it acts as an agonist at the somatodendritic 5HT1A autoreceptors found in the dorsal raphe serotonergic neurons. The latter has major projections to the prefrontal cortex and decreased serotonergic firing mediated by the activation of 5HT1A autoreceptors, in turn, causes an increase in the dopaminergic tone. Working through this mechanism, aripiprazole may improve cognitive and negative symptoms in bipolar spectrum disorders and schizophrenia. In this vein, brexpiprazole and aripiprazole have been more aptly described as “serotonin-dopamine activity modulators” [18]. Further, aripiprazole has been shown to act as an agonist at 5HT2C receptors and this action has been regarded as advantageous in decreasing appetite and avoiding antipsychotic-induced weight gain. 5HT2A antagonism is a property that aripiprazole shares with other SGAs and the ramifications of this interaction embrace a reduction in EPSE during therapy with these agents. Finally, it only moderately blocks the alpha 1 adrenergic and histamine H1 receptors, which may account for the low incidence of orthostatic hypotension and sedation with the use of this compound. 

### Tolerability and safety 

Data from short and long-term studies in bipolar subjects indicates that aripiprazole has a good tolerability and safety profile. During the acute three-week monotherapy trials, the most commonly noted adverse effects versus placebo were akathisia (13% vs. 4%), sedation (8% vs. 3%), restlessness (6% vs. 3%), tremor (6% vs. 3%), and EPSE (5% vs. 2%) [19-20]. In a six-week study of aripiprazole adjunctive to mood stabilizers, akathisia (19% v. 5%), insomnia (8% v. 4%), and extrapyramidal side effects (5% vs. 1%) were most frequently described [21]. Two recurrence prevention trials for 52 and 100 weeks showed tremor, akathisia, hypertension, and dry mouth to be the common side effects [22-23]. Across the long-term trials, the most common treatment-emergent adverse events that lead to treatment discontinuation in aripiprazole recipients were mania (1%) or manic reaction (7%), akathisia (5%), and tremor (2%).

Extrapyramidal Side Effects

Apparently because of aripiprazole’s low intrinsic activity and partial agonism at D2R, there is a sustained stimulating effect of the endogenous ligand, dopamine, in the nigrostriatal pathway accounting for fewer EPSE with its use. The most commonly reported adverse events are akathisia and tremor while the rest of the EPSE-like dystonias, pseudo-parkinsonism, and tardive dyskinesias are conspicuously uncommon. When EPSE occur, these are generally of mild to moderate nature, translating into a low discontinuation rate for aripiprazole because of these side-effects.

Metabolic Effects

Bipolar disorder is viewed as a systemic illness with such manifestations as obesity, serum lipid abnormalities, and diabetes. Many atypical antipsychotics have the liability to cause or aggravate the metabolic syndrome, which is marked by increased body mass index, dyslipidemia, and glucose intolerance. According to a comprehensive literature review, among the SGAs, clozapine, olanzapine, quetiapine, and risperidone emerge as the worst offenders, whereas aripiprazole appears to have the fewest adverse metabolic effects [24]. The safety of aripiprazole in bipolar subjects is borne out by a post-hoc analysis of a 26-week study, which shows rates of metabolic syndrome with aripiprazole comparable to placebo [25]. According to the data from controlled short and long-term studies, there are no significant negative effects of aripiprazole mono or adjunctive therapy on such parameters as serum cholesterol, triglycerides, or glucose.

Effect on Prolactin Secretion

The discharge of dopamine from the hypothalamus is a signal for the anterior pituitary gland to prohibit the release of prolactin, and antipsychotics, which strongly bind to D2 receptors like haloperidol and risperidone, let go of this control by acting as antagonists. Contrarily, aripiprazole, a partial agonist at the D2R, appears to facilitate dopamine transmission in the tuberoinfundibular pathway and prevents hyperprolactinemia from occurring. Raised serum prolactin is associated with such untoward effects as sexual dysfunction, amenorrhea, galactorrhea, and bone demineralization. There is evidence that aripiprazole therapy may perhaps be associated with an overall decline in the serum prolactin level in bipolar patients who switch from other antipsychotics to this medication [26].

Effect on Electrocardiogram (ECG)

Some psychotropic medications, including antipsychotics such as thioridazine and ziprasidone, are associated with prolongation of the corrected QT (QTc) interval, which can potentially lead to such lethal arrhythmias as torsades de pointes. As far as aripiprazole is concerned, evidence from studies in bipolar patients shows that it has no such untoward effect and does not cause QTc prolongation [26].

### Studies in bipolar mania

Acute Manic Phase Studies

The efficacy of aripiprazole monotherapy in the treatment of acute mania has been studied in at least four RCTs. According to the existing literature, the most recent study was a multi-center, double-blind trial of Japanese patients, which investigated the efficacy and safety of aripiprazole (24 mg/day) versus placebo for three weeks. Statistically significant mean improvements were noted from Day 4 onwards to Day 21 (-11.3 vs. -5.3 on Young Mania Rating Scale [YMRS]) at endpoint [27]. These results were in line with two earlier key trials of aripiprazole, 30 mg, versus placebo conducted mainly in Caucasian populations [19-20]. In an RCT, Bipolar I patients with acute manic or mixed episodes were randomized to two fixed doses of aripiprazole (15 or 30 mg/d) or placebo. In this three-week trial, both of the active agent groups were not statistically better than placebo on the primary efficacy measure of YMRS at endpoint [28]. 

In a well-powered trial, both aripiprazole and haloperidol showed a significantly greater mean change in the YMRS compared to placebo at Week 3 with equivalent improvements for the two medications at Week 12 [29]. In another study evaluating aripiprazole, lithium, and placebo for three and 12-week periods, aripiprazole showed significant improvement in the mean YMRS score within two days. This improvement carried on over three weeks and was sustained over 12 weeks in an analogous manner for both drugs [30]. In another trial, more acutely manic patients were in remission and taking aripiprazole compared to haloperidol at the study endpoint at Week 12 [31]. In summary, in these trials, which compared aripiprazole (doses of 15-30 mg) to established standard antimanic treatments, there was an equivalent efficacy of the active agents. Additionally, using YMRS scores as the benchmark, corresponding early and continued separation from placebo was noted and remission rates were achieved at three and 12 weeks, with the advantage of proportionately lower drop-outs in subjects taking aripiprazole. 

In an RCT, aripiprazole showed additional benefits adjunctive to lithium or valproate in acute manic and mixed episodes. Bipolar I patients with acute mania partially nonresponsive to the mood stabilizer alone and receiving combined treatment with aripiprazole and one of the mood stabilizers demonstrated a significantly higher reduction of their baseline YMRS score at Week 6 versus placebo (at study endpoint: -13.3 for aripiprazole, -10.7 for placebo). In this trial, similar to the monotherapy studies, significant improvement in YMRS total score was noted with aripiprazole versus placebo from Week 1 onward [21]. In a study, both adjunctive aripiprazole and haloperidol with the first-line mood stabilizer, divalproex sodium, were equally efficacious in acute bipolar mania; the novel antipsychotic was associated with fewer EPSE as compared to haloperidol but caused greater weight gain and sedation [32]. In subjects aged 10 to 17 years and presenting with acute manic or mixed episodes, aripiprazole monotherapy was statistically superior to placebo in inducing remission, was well-tolerated, and did not result in any significant weight gain [33]. A post-hoc analysis of two short-term, three-week monotherapy trials of subjects with BD Type I experiencing acute manic or mixed episodes is instrumental in elucidating the efficacy of this medication across a wide range of illness characteristics associated with treatment resistance. The data was stratified according to the severity of manic symptoms, the presence of depressive features, psychosis, and history of rapid cycling. The salutary effects of aripiprazole therapy were seen in these different subsets of cases with severe disease attributes, epitomizing that this agent has an important place in the treatment of bipolar diathesis [34]. Finally, while this article was in revision, a published meta-analysis showed that in both controlled and real-world settings, aripiprazole was superior to placebo and equal to active comparators in acute and stabilization phases of bipolar mania. Its safety profile was similar to other antipsychotics when considering such side effects as sedation, akathisia, EPSE, and weight gain but had a notably lower tendency to cause hyperprolactinemia [35].  

The data presented above is further explained in Table 2. 

**Table 2:** RCTs of Aripiprazole in Acute Mania Abbreviations: ARI – aripiprazole, DB – double blind, EPSE – extrapyramidal side-effects, HAL – haloperidol, LI – lithium, PLB – placebo, RCT – randomized controlled trial, VAL – valproate, YMRS – Young Mania Rating Scale.

Study	Rescue medications	Design	Subjects	Primary outcome
Keck, et al., 2003 [19]	Lorazepam, benztropine	DB, 3-week primary endpoint, ARI 30 mg/day fixed-dose (could be reduced to 15 mg/day)	262 subjects with acute manic or mixed episodes, mean YMRS at baseline 28.2 (ARI) and 29.7 (PLB)	YMRS reduction: -8.2 (ARI), -3.4 (PLB), *p* ≤ 0.002
Sachs, et al., 2006 [20]	Lorazepam, benztropine	DB, 3-week primary endpoint, ARI 30 mg/day fixed-dose (could be reduced to 15 mg/day)	272 subjects with acute manic or mixed episodes, mean YMRS at baseline 28.8 (ARI) and 28.5 (PLB)	YMRS reduction: -12.5 (ARI), -7.2 (PLB), *p* ≤ 0.001
Kanba, et al., 2014 [27]	Short-acting benzodiazepines, biperiden	DB, 3-week primary endpoint, ARI 24 mg/day fixed-dose (could be reduced to 12 mg/day)	258 subjects with acute manic or mixed episodes, mean YMRS at baseline 28.3 (ARI) and 28.0 (PLB)	YMRS reduction: -11.3 (ARI), -5.3 (PLB), *p* ≤ 0.001
El Mallakh, et al., 2010 [28]	Lorazepam, benztropine	DB, 3-week primary endpoint. ARI 30 mg/day or 15 mg/day fixed-dose	401 subjects with acute manic or mixed episodes, mean YMRS at baseline 27.9 (ARI 15 mg), 27.3 (ARI 30 mg), 28.3 (PLB)	YMRS reduction: - 10.0 (ARI 15 mg), -10.8 (ARI 30 mg), - 10.1 (PLB), *p* = not significant
Young, et al., 2009 [29]	Benzodiazepines, anticholinergics for EPSE, propranolol for tremor or akathisia	DB, 3-week primary endpoint, ARI 15-30 mg/day, HAL 5-15 mg/day flexible dosing. DB continuation of ARI and HAL until Week 12 (secondary endpoint)	485 subjects with acute manic or mixed episodes, mean YMRS at baseline 28.4 (ARI), 28.0 (HAL), 28.8 (PLB)	YMRS reduction at week 3: -12.0 (ARI), -12.8 (HAL), -9.7 (PLB). *p* = 0.039 for ARI and *p* ≤ 0.005 for HAL
Keck, et al., 2009 [30]	Benzodiazepines, benztropine, propranolol	DB, 3-week primary endpoint, ARI 15-30 mg/day, LI 900-1500 mg/day flexible dosing. DB continuation of ARI and LI until Week 12 (secondary endpoint)	480 subjects with acute manic or mixed episodes, mean YMRS at baseline 28.5 (ARI), 29.4 (LI), 28.9 (PLB)	YMRS reduction at week 3: -12.6 (ARI), -12.0 (LI), -9.0 (PLB). *p *≤ 0.001 for ARI and *p* = 0.005 for LI
Vieta, et al., 2008 [21]	Benzodiazepines, anticholinergics, propranolol	DB, 6-week primary endpoint. ARI adjustable dose 30 mg/day or 15 mg/day or PLB add on to LI or VAL. Partial non-responders with a YMRS ≥ 16 after 2 weeks of LI or VAL with therapeutic plasma levels	384 subjects with acute manic or mixed episodes, mean YMRS at baseline 23.1 (ARI), 22.7 (PLB)	YMRS reduction: -13.3 (ARI), -10.7 (PLB), *p* ≤ 0.01

Continuation Phase Studies in Patients with Index Manic or Mixed Episodes

The safety and efficacy of aripiprazole have been studied in RCTs in the continuation phase following treatment of acute manic or mixed episodes in Bipolar I disorder. In a placebo-controlled monotherapy trial, hospitalized bipolar patients with manic or mixed episodes were initially given open-label aripiprazole (15 or 30 mg/d). Those who achieved sustained remission (YMRS ≤ 10; MADRS ≤ 13) for at least six weeks were assigned to either placebo or aripiprazole in a double blind way and followed for the total study duration of 26 weeks. It was determined that the active treatment (both doses) was statistically significant compared to placebo in delaying time to manic, but not depressive, relapse and was relatively well tolerated with a low incidence of side effects [36].

A 52-week trial evaluated the safety and efficacy of aripiprazole + lithium or valproate versus placebo + lithium or valproate in manic subjects who had an inadequate response to mood stabilizer monotherapy during at least two weeks of treatment. Aripiprazole was added in a non-double blind mode and those patients who became stable for 12 consecutive weeks (YMRS and MADRS scores ≤ 12) were randomized to the active drug or placebo and followed in a double-blind manner for a total of 52 weeks. The results showed that, evaluated against the placebo, significantly more patients who responded to add-on aripiprazole in the acute manic or mixed phase remained relapse-free with the active drug during the continuation phase. This finding suggested that there was a long-term benefit in continuing aripiprazole adjunctive to the first-line mood stabilizers for relapse prevention of manic and mixed episodes, once sustained remission was achieved with the combination regimen in Bipolar I disorder patients [22]. A post-hoc analysis of a 52-week double-blind, placebo-controlled trial of adjunctive aripiprazole and first-line mood stabilizers in Bipolar I patients with initial manic or mixed episodes showed that this agent was more effective in preventing relapses in those with index manic, as compared to mixed episodes [37].

In a 52-week study, completers of a six-week double-blind comparison of adjunctive aripiprazole versus placebo in bipolar mania who were only partially responsive to previous lithium or valproate monotherapy could enter a 46-week extension with open-label aripiprazole + lithium or aripiprazole + valproate. Using the last observation carried forward to account for drop-outs, it was noticed that statistically significant improvement from baseline over the 52 weeks occurred with aripiprazole + lithium/valproate on the mean YMRS and Montgomery-Asberg Depression Rating Scale (MADRS) total scores. In summary, this open-label study demonstrated that long-term aripiprazole in conjunction with traditional mood stabilizers was efficacious in the prophylaxis of manic or mixed episodes, maintained the functioning of bipolar patients, and had a good safety and tolerability profile [38]. A continuation phase RCT in Korean patients could not replicate these findings. In this 24-week study, adjunctive aripiprazole and lithium or valproate were not statistically better than placebo + mood stabilizer in preventing affective recurrences [39]. In a long-term trial, aripiprazole + lamotrigine was not significantly superior to placebo + lamotrigine in delaying time to affective relapse in Bipolar I patients [40].

Lastly, in a long-term 52-week study, aripiprazole and lithium were analyzed head-to-head in a double-blind manner. In the stabilization phase, Bipolar I disorder subjects with initial manic or mixed episodes were treated double-blind with the above-mentioned medications for 12 weeks. Those who achieved sustained remission (YMRS and MADRS scores ≤ 12), were followed in the same manner for a further period of 40 weeks. Statistics were drawn on observed cases only, and although there was a high attrition rate, the completers of the study had equivalent benefit in the prevention of manic and mixed episodes. In contrast to lithium, data showed that aripiprazole was better tolerated with fewer and less severe adverse events [41].

 A summary of the key long-term trials of aripiprazole in BD is provided in Table 3. 

**Table 3:** Key Long-Term Aripiprazole Studies in Mania Abbreviations: AE – adverse events, ARI – aripiprazole, BD – bipolar disorder, CI – confidence interval, DB – double blind, LI – lithium, LOCF – last observation carried forward, MADRS – Montgomery-Asberg Depression Rating Scale, PLB – placebo, VAL – valproate, YMRS – Young Mania Rating Scale

Study	Design	Subjects	Outcome	Adverse events	Conclusion
El-Mallakh, et al., 2012 [41]	DB. After initial randomization to 12 weeks of LI vs. ARI monotherapy, patients continued to receive either ARI 15-30 mg/day or LI 900, 1200 or 1500 mg/day for further 40 weeks.	66 patients with index manic or mixed episodes entered the extension phase. Only 20 completed the entire phase (ARI n = 7; LI n = 13). Efficacy endpoints included adjusted mean change from baseline to week 52 in YMRS and MADRS total scores.	Remission defined as YMRS total score ≤ 12. Significant improvement that occurred over the first 12 weeks was maintained over the 40 weeks of continuation.	ARI – akathisia, headache, somnolence, anxiety, and nasopharyngitis (all 8%). LI – insomnia (15.8%), headache (13.2%), diarrhea (13.2%), and vomiting (10.5%). Mean weight change +2.71 kg for LI and +5.66 kg for ARI, *p*= 0.46	ARI monotherapy equivalently efficacious to LI for extended treatment of BD patients with index manic or mixed episodes. ARI better tolerated than LI in the long term.
Marcus, et al., 2011 [22]	Patients with a manic or mixed episode received LI or VAL for at least 2 weeks. Those with an inadequate response [YMRS total score ≥ 16 and ≤ 35% decrease from baseline at week 2] received single-blind ARI + MS. Patients who achieved stability [YMRS and MADRS score ≤ 12] for 12 consecutive weeks were randomized to DB ARI (10-30 mg/day) or PLB + LI/VAL for 52 weeks.	A total of 337 patients were randomized to ARI + LI/VAL (n = 168) or PLB + LI/VAL (n = 169).	The Kaplan-Meier relapse rate at 52 weeks was 17% with ARI + LI/VAL and 29% with PLB + LI/VAL; hazard ratio = 0.54 (95% CI: 0.33-0.89; log-rank *p* = 0.014)	The most common AE ≥ 5% (ARI + LI/VAL vs. PLB + LI/VAL) were headache (13.2% vs. 10.8%), weight increase (9.0% vs. 6.6%), tremor (6.0% vs. 2.4%), and insomnia (5.4% vs. 9.6%).	Continuation of ARI + LI/VAL treatment increased time to relapse to any mood episode compared with LI or VAL monotherapy and was relatively well tolerated during the 1-year study period. These findings suggest that there is long-term benefit in continuing ARI adjunctive to MS after sustained remission is achieved.
Vieta, et al., 2010 [38]	DB with open label extension. BD patients with manic or mixed episodes were treated with open-label LI or VAL. Those with inadequate response were randomized to ARI + LI/VAL or PLB + LI/VAL for 6 weeks in a DB manner. Completers of the 6-week DB trial could enter a 46-week extension with open-label ARI + LI or ARI + VAL.	In total, 283 (ARI + LI n = 108; ARI + VAL n = 175) patients entered the 46-week open label extension. Out of them, 146 (ARI + LI n = 55; ARI + VAL n = 91) completed the trial.	Significant improvements from baseline over the 52 weeks occurred with ARI + LI and ARI + VAL (LOCF). Mean YMRS total score changes ARI + LI -16.5 (95% CI; -18.1 to -14.8) and ARI + VAL -17.6 (95% CI; -18.9 to -16.3), both *p* < 0.001 versus baseline. Mean MADRS total score chances ARI + LI -1.7 (95% CI; -3.3 to -0.1, *p* < 0.05 versus baseline) and ARI + VAL -2.7 (95% CI; -4.0 to -1.4, *p* < 0.001 vs. baseline).	Frequently reported AE with ARI + LI vs. ARI + VAL: tremor (17.0% vs. 12.1%), akathisia (6.6% vs. 8.6%), headache (6.6% vs. 4.0%), insomnia (9.4% vs. 10.3%), depression (7.5% vs. 9.2%) and weight increase (11.3% vs. 8.6%). The majority of new onset akathisia and insomnia occurred early. Mean weight change from DB endpoint to week 46 (LOCF) was 2.3 (0.6) kg with ARI + LI and 2.0 (0.4) kg with ARI + VAL.	Long-term ARI adjunctive to LI/VAL in bipolar mania was safe and well tolerated. Improvements in manic symptoms and functioning were maintained. ARI, adjunctive to either LI or VAL appeared to be equally safe and effective combinations for the treatment of BD.
Keck, et al., 2007 [23]	DB. BD patients with recent episode manic or mixed received open-label ARI 15-30 mg/day for 6 to 18 weeks. Patients who achieved stabilization (YMRS score ≤ 10; MADRS score ≤ 13 for 6 consecutive weeks) were randomized to DB treatment with ARI or PLB for 26 weeks. The primary endpoint was time to relapse for any mood episode. Patients who completed the 26-week stabilization and maintained remission were continued in a DB manner with ARI or PLB for an additional 74 weeks.	A total of 161 patients met the stabilization criteria and were randomly assigned to ARI (n = 78) and PLB (n = 83).	At 100 weeks, time to relapse was significantly longer for ARI than PLB (hazard ratio = 0.53 [95% CI = 0.32 to 0.87], *p* = 0.011). ARI was superior to PLB in delaying time to manic relapse (hazard ratio = 0.35 [95% CI = 0.16 to 0.75], *p* = 0.005). However, no significant differences were observed in time to depressive relapse (hazard ratio = 0.81 [95% CI = 0.36 to 1.81], *p* = 0.602)	AE reported during 100 weeks of treatment with ARI versus PLB (≥ 5% incidence and twice placebo rate) were tremor, akathisia, dry mouth, hypertension, weight gain, vaginitis, abnormal thinking, pharyngitis, and flu syndrome. Mean weight change from baseline to 100 weeks (LOCF) was + 0.4 +/-0.8 kg with ARI and -1.9 +/-0.8 kg with PLB.	Over a 100-week treatment period, ARI monotherapy was effective for relapse prevention of manic or mixed episodes in BD patients who were initially stabilized on ARI for 6 consecutive weeks. ARI maintained a good safety and tolerability profile during the 100-week trial period.

Maintenance Study in Patients with Initial Manic or Mixed Episode

A 100-week aripiprazole monotherapy study was conducted in a placebo-controlled, double-blind style to assess the safety and efficacy of the drug in preventing relapse of mood episodes in Bipolar I disorder patients. Bipolar subjects with a recent manic or mixed episode received open-label aripiprazole, 15 or 30 mg per day, for six to 18 weeks. Patients achieving stabilization (YMRS score ≤ 10, MADRS score ≤ 13 for six consecutive weeks) entered the double-blind phase, at which point they were randomly assigned to treatment with aripiprazole or placebo for 26 weeks. The primary endpoint was time to relapse for any mood episode. Patients who remained in remission during the 26-week stabilization period continued in a double-blind fashion with aripiprazole or placebo for an additional 74 weeks and were monitored for relapse, efficacy, and tolerability. In total, 161 patients met the stabilization criteria and were randomly assigned to aripiprazole (N=78) or placebo (N=83). At 100 weeks, time to relapse was significantly longer with aripiprazole than placebo; the active drug was superior to placebo in delaying time to manic relapse. However, no significant differences were observed in time to depressive relapse. Adverse events for aripiprazole occurring in more than 5% of the subjects included tremor, akathisia, dry mouth, flu syndrome, and mild weight gain, among others. In conclusion, over a 100-week treatment period, aripiprazole monotherapy was effective for manic relapse prevention and maintained a good safety and tolerability profile [23]. In a post-hoc analysis of the 100-week maintenance study, patients with rapid cycling were identified and efficacy of aripiprazole monotherapy versus placebo was examined. In this group of cases, the active drug was statistically superior to no therapy in preventing affective recurrences in an otherwise recalcitrant manifestation of BD [42].

Clinical Implications of Acute and Long-term Aripiprazole Studies in Bipolar Mania

In view of the efficacy and safety data presented above, aripiprazole should be considered a first-line agent in the treatment of manic or mixed episodes in Bipolar I disorder patients. It can be preferentially administered in both new onset and recurrent mania, either as monotherapy or as an add-on to mood stabilizers, and may be used favorably in the acute and maintenance phases with the manic polarity of illness. Further, when patients experiencing a manic or mixed episode have less than optimal response to monotherapy with conventional mood stabilizers, aripiprazole is a valid choice as an adjunctive medication to induce remission.

Akin to schizophrenia, it is a switch option in bipolar patients who poorly put up with other antipsychotics due to side effects and should be preferred in the long term treatment of such cases to promote compliance. As demonstrated in acute and long-term RCTs, it has notably few adverse effects and is well tolerated as mono and adjunctive therapy, with lower discontinuation rates as compared to typical antipsychotics, for example, haloperidol. It should be used in preference to agents like olanzapine in patients with high body mass index, dyslipidemia, and glucose intolerance. When a metabolic syndrome is induced during the ongoing treatment of bipolar subjects with other SGAs, aripiprazole is a valid alternate medication. Correspondingly, it can be utilized as a switch option in patients who develop hyperprolactinemia, EPSE, QTc prolongation, drowsiness, and sedation with the use of either typical or second generation antipsychotics in the course of treatment of BD. 

### Third generation antipsychotics in the treatment of  bipolar depression

Although the distinctive clinical feature of bipolar disorder is the abnormally elevated mood seen in manic exacerbations, it does not usually make up the most common affective state of the patients. The majority of bipolar patients spend much more time suffering from depressive manifestations, which may present as major depressive episodes, sub-syndromal symptoms, or mixed episodes [43]. Depressive states are often comorbid with other neuropsychiatric conditions like anxiety spectrum disorders and are the major source of morbidity and mortality in BD. Depressive symptoms frequently show resistance to the usual mood stabilizers and are incriminated in refractoriness and chronicity of the illness. Used either as monotherapy or adjunctively to mood stabilizers, third generation antipsychotics are emerging as a credible treatment option for bipolar depression. The latest positive published trial in this respect is with cariprazine, a novel antipsychotic that acts as a partial agonist at D2R and D3R and, like aripiprazole and brexpiprazole, is a multipurpose psychotherapeutic agent [44]. The next three sections evaluate the data regarding the efficacy of aripiprazole in the acute treatment and prophylaxis of bipolar depression. 

Randomized, Controlled Trials of Aripiprazole in Bipolar I Depression

Two matching eight-week, multicenter RCTs with placebo control (CN 138-096 [Study 1] and CN 138-146 [Study 2]) were carried out to assess the efficacy and safety of aripiprazole in outpatients with Bipolar I disorder experiencing a major depressive episode without psychotic features. Patients were randomized to placebo or aripiprazole started at 10 mg per day, then dosed in a versatile style at 5-30 mg/d based on clinical effect and tolerability. The main end point was mean change from baseline to Week 8 in the MADRS total score. The last observation carried forward was utilized to statistically account for subjects who dropped out of the trials prematurely. In Studies 1 and 2, respectively, 186 and 187 patients were randomized to aripiprazole, and 188 and 188 to placebo. Although numerically significant distinctions were detected during Weeks 1 to 6, aripiprazole did not pull off statistical meaning versus placebo at Week 8 in either study. This was made evident as an inconsequential reduction in MADRS total score from the baseline, which was the most important outcome measure. The key secondary endpoint was the Clinical Global Impressions Bipolar Version Severity of Illness-Depression score, and although the active agent showed initial statistical parting, it was not found to be better than placebo at Week 8 in this measure either. Aripiprazole was associated with a higher incidence of akathisia, insomnia, nausea, fatigue, restlessness, and dry mouth versus placebo. More patients stopped taking aripiprazole versus placebo in Study 1 (46.8% vs. 35.1%) and Study 2 (41.2% vs. 29.8%). Aripiprazole monotherapy, as dosed in this design, was not significantly more effective than placebo in the treatment of bipolar depression at study end point of Week 8 [45].

In the management of acute episodes in a major depressive disorder, aripiprazole is an established treatment as an add-on to standard antidepressants. However, this is not the case with acute bipolar depression as demonstrated in an RCT in which aripiprazole failed to separate from placebo on the primary efficacy measure of Hamilton Rating Scale for Depression when used with open adjunctive citalopram [46].

Post-hoc Analysis of Data from Aripiprazole Monotherapy Trials in Acute Bipolar I Depression

A pooled, post-hoc examination of data from the above-mentioned two randomized, controlled trials was undertaken and the outcome of aripiprazole monotherapy versus placebo was assessed based on the initial severity of depressive symptoms of the study subjects. This was achieved by categorizing cases as severely (Bech-6 total score > 15) or less severely depressed (Bech-6 total score < 15) using sub-sets of Bech-Rafaelsen scale. Efficacy was measured by mean variations in MADRS total and MADRS-6 subscale scores from the initiation of the trials to the endpoint at Week 8 using a mixed model, repeated measures analysis. In this manner, a sum of 133 patients (n = 62 on the active drug) was found to be severely depressed and 612 patients (n = 309 on aripiprazole) as less severely depressed. At Week 8, the mean MADRS total score reduction for severely depressed patients receiving aripiprazole compared with placebo was -19.4 vs. -15.4 (*p* = 0.14), whereas MADRS-6 subscale score reduction for patients receiving aripiprazole compared with placebo was -13.8 vs. -10.3 (*p* = 0.07). Adverse event profiles were similar between the two severity groups. The sum of the analysis was that improvement in essential depressive symptoms occurred with flexibly dosed aripiprazole monotherapy in subjects with more severe major depressive episodes than those with less severe manifestations in Bipolar I disorder [47].

Prevention of Depressive Relapse

Compared to placebo, aripiprazole monotherapy was shown in a maintenance study to delay time to manic relapse but not for depressive relapse after 26 and 100 weeks of treatment [23]. The inability of aripiprazole to reduce the time to depressive relapse in this study could be due to the enrollment of patients with index manic/mixed episodes, a manic polarity of illness, and consequently, lower occurrence of depressive relapses. Another long-term trial comparing aripiprazole and lithium in a head-to-head manner showed that both drugs were equally efficacious in preventing affective recurrences of manic and mixed type but the former agent did not obviate depressive relapses [41]. A 52-week trial comparing aripiprazole or placebo adjunctive to conventional mood stabilizers showed that the active drug, plus lithium or divalproex, was more effective in manic than depressive relapse prevention [22]. Although data does not support the efficacy of aripiprazole in precluding depressive relapses, there is a need for more and better-designed studies in this regard. One strategy may be to enroll bipolar patients with index depressive episodes in long-term aripiprazole trials, either as monotherapy or in conjunction with mood stabilizers. Further, when proposing protocols for novel psychotherapeutic agents in bipolar depression, exclusive reliance on efficacy measures like MADRS may not portray the whole picture. There is also an argument for including other parameters that assess general functioning, reduction in comorbidities, and decline in overall symptomatic burden [48]. It must not be forgotten that BD is linked to numerous comorbidities, chief among which are substance use disorders and anxiety spectrum conditions. Aripiprazole is a multifunction drug, and in addition to targeting core affective symptoms, it has important implications for the treatment of associated neuropsychiatric comorbidities in bipolar spectrum disorders. 

Aripiprazole in the Adjunctive Treatment of Major Depressive Disorder (MDD)

MDD is a major psychiatric ailment and shares phenotypic, pathophysiologic, and prognostic characteristics with bipolar disorder. It is a recurrent condition and is often refractory to treatment with the usual antidepressant medications, resulting in continued morbidity in spite of optimal therapy with the latter agents. In this event, there is an enduring need for psychotherapeutic drugs, which are truly efficacious in MDD and can stall the vicious cycle of deterioration in the biopsychosocial domains. RCTs of aripiprazole have been conducted in this disorder, mainly as an adjunct to first-line antidepressants and salutary affects statistically demonstrated with standardized measures [49]. This is of significance, as MDD is a recalcitrant condition with neuroprogressive features, and aripiprazole, being the prototypical third generation antipsychotic, has opened fresh avenues in the overall treatment of this disorder. 

### New directions and emerging therapies for bipolar and related disorders

**Figure 4:**
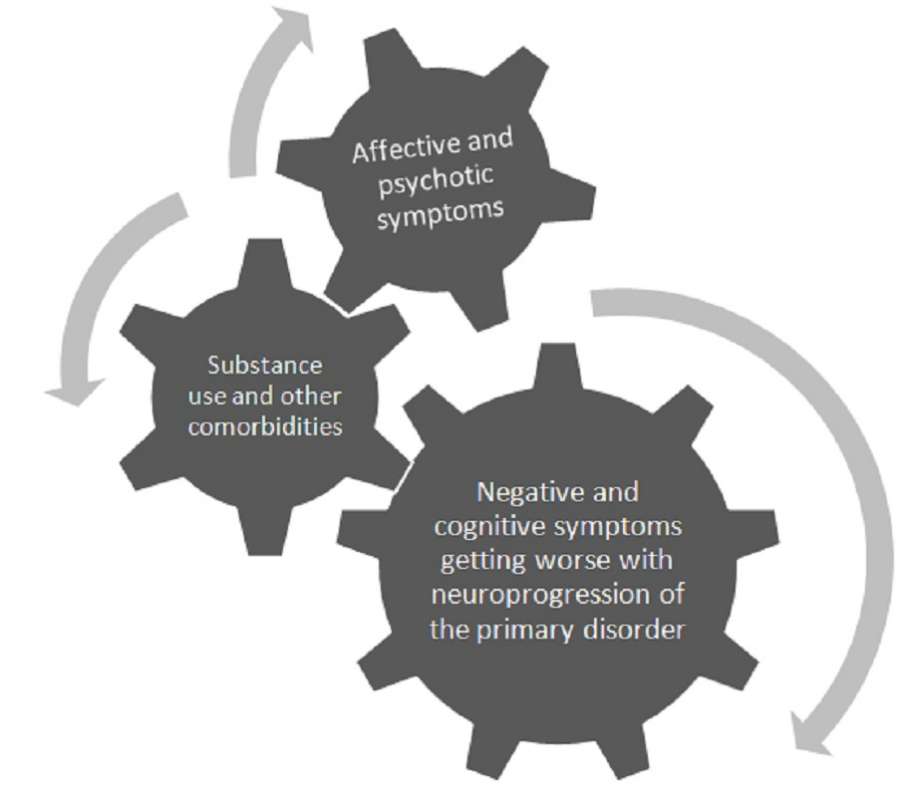
Targeting neuropsychiatric symptoms in major mental disorders Principal psychiatric disorders share the common feature of neuroprogression, which implies that there is increasing biopsychosocial impairment as the illness advances. Novel agents like aripiprazole, brexpiprazole, and cariprazine have the potential to treat various disease manifestations and be of value in affective, psychotic, cognitive, and negative symptoms.

Partial agonism, functional selectivity, and serotonin-dopamine activity modulation are the new pharmacodynamic principles in the treatment of major psychiatric disorders, including bipolar spectrum disorders. In the case of aripiprazole, evidence from controlled trials supports its usefulness in acute manic and mixed episodes, rapid cycling, psychotic mania, and prevention of manic recurrences in Bipolar I disorder. It is an FDA approved therapy for schizophrenia and adjunctive treatment of MDD, which expounds the fact that aripiprazole is a versatile psychotherapeutic agent. As a further advancement in drug development, two new related compounds, brexpiprazole and cariprazine, have won FDA endorsement, while a number of other molecules are in different stages of experimentation. These are the latest “third generation antipsychotics” which are, in essence, multipurpose medications and have the promise of alleviating hitherto recalcitrant manifestations like cognitive and negative deficits associated with neuroprogressive psychiatric disorders [50]. As the deficit symptoms are responsible for the true burden of principal psychiatric diseases, the recent paradigm shift in pharmacotherapy is opening fresh avenues in the treatment of these conditions. It is beyond the scope of this manuscript to go into the details of published RCTs and ongoing studies of brexpiprazole and cariprazine, but novel antipsychotics hold the potential of improving the overall prognosis of patients with major psychiatric ailments. Table 4 gives FDA approved indications of aripiprazole, brexpiprazole, and cariprazine at the time of the writing of this manuscript. Figure 4 illustrates the therapeutic prospects of this group of medications. 

**Table 4:** FDA Approved Indications of Aripiprazole, Brexpiprazole and Cariprazine Abbreviations: FDA – Food and Drug Administration; MDD – major depressive disorder; MS – mood stabilizers; RCT – randomized controlled trial

Indications	Aripiprazole	Brexpiprazole	Cariprazine
Schizophrenia	Yes	Yes	Yes
Manic/mixed episodes of Bipolar I disorder	Yes	No	Yes
Maintenance treatment of Bipolar I disorder	Yes, as an adjunct to MS	No	No
Bipolar I depression	No (negative RCT)	No	No (positive RCT)
MDD	Yes, as an adjunct	Yes, as an adjunct	No

## Conclusions

Bipolar disorder is a highly disabling condition with a large number of patients remaining symptomatic either because they are refractory to the traditional mood-stabilizing medications or cannot tolerate these due to adverse effects. There is a pressing need for new and better drugs, which are efficacious in treating as well as preventing mood episodes in these patients. Aripiprazole is the first among the unique group of novel antipsychotics that is well studied in all phases of BD and controlled trials have been very helpful in delineating its place in the treatment of this chronic, life-long disorder. It is particularly efficacious in managing patients with the manic polarity of illness and is safe and well tolerated by the majority of treated subjects. Although controlled trials have not proven its worth in acute or recurrent bipolar depression, it may have a place in treating bipolar spectrum disorder patients with substance use and other comorbidities. Currently, it is highly recommended for the management of acute and recurrent mania in Bipolar I disorder. The same appears to be true for mixed episodes, rapid cycling, and psychotic mania. Considering its value in the treatment of anxiety spectrum and addictive disorders, it can be efficacious in bipolar subjects with these concomitant conditions. It is also the preferred agent in patients with metabolic abnormalities and those who are intolerant of other antipsychotics due to the latter’s side effect load.
